# APRIL, a proliferation-inducing ligand, as a potential marker of lupus nephritis

**DOI:** 10.1186/ar4095

**Published:** 2012-11-21

**Authors:** Worapot Treamtrakanpon, Pornpen Tantivitayakul, Thitima Benjachat, Poorichaya Somparn, Wipawee Kittikowit, Somchai Eiam-ong, Asada Leelahavanichkul, Nattiya Hirankarn, Yingyos Avihingsanon

**Affiliations:** 1 Division of Nephrology, Department of Medicine, Faculty of Medicine, Chulalongkorn University, 1873 Rama IV Road, Pathumwan, Bangkok, Thailand, 10330; 2 Lupus Research Unit, Department of Medicine, Faculty of Medicine, Chulalongkorn University, 1873 Rama IV Road, Pathumwan, Bangkok, Thailand, 10330; 3 Department of Pathology, Faculty of Medicine, Chulalongkorn University, 1873 Rama IV Road, Pathumwan, Bangkok, Thailand, 10330; 4 Department of Microbiology; Faculty of Medicine, Chulalongkorn University, 1873 Rama IV Road, Pathumwan, Bangkok, Thailand, 10330; 5 Biomedical Science, Interdisciplinary Program, Graduate School, Chulalongkorn University, 254 Phayathai Road, Pathumwan, Bangkok, Thailand, 10330; 6 Department of Oral Microbiology, Faculty of Dentistry, Mahidol University, 6 Yothe Road, Rajthevee, Bangkok, Thailand, 10400

## Abstract

**Introduction:**

BLyS and APRIL are cytokines from the tumor necrosis factor family which play an important role in systemic lupus erythematosus (SLE). Previous works suggested an association between both molecules and SLE disease activity although their correlation with lupus nephritis is not known. We therefore assessed serum BLyS and APRIL in active lupus nephritis patients.

**Methods:**

Serum samples from active lupus nephritis and at 6 months post-treatment were obtained. Serum levels of BLyS and APRIL (*n *= 47) as well as renal mRNA expression were measured. Serum levels of both molecules and clinical data (*n *= 27) were available at 6 months follow-up. All biopsy-proven lupus nephritis patients were treated with similar immunosuppressive drugs.

**Results:**

Serum levels of APRIL were associated with proteinuria (Rs = 0.44, *P *value < 0.01) and degree of histological activity (Rs = 0.34; *P *value < 0.05) whereas BLyS levels were associated with complement levels (Rs = 0.46; *P *value < 0.01) and dosage of immunosuppressant. Interestingly, serum APRIL as well as its intrarenal mRNA levels were associated with resistance to treatment. From the receiver operating characteristic (ROC) analysis, high levels (> 4 ng/mL) of serum APRIL predicted treatment failure with a positive predictive value of 93 percent.

**Conclusion:**

APRIL could be a potential biomarker for predicting difficult-to-treat cases of lupus nephritis.

## Introduction

B cell regulation plays an important role in the pathogenesis of systemic lupus erythematosus (SLE) and lupus nephritis (LN) [1,2]. Many approaches have targeted blocking specific pathways of B cell activation for the treatment of the disease. It has also been shown that when B cells were depleted, this could ameliorate the progression of the disease [3-7]. Anti-CD20 monoclonal antibody was able to normalize autoantibodies and serum complement levels even though its clinical efficacy for lupus nephritis was a failure in clinical trials [8]. However, selection criteria for participants in the trials are vital, and this may partly be the explanation for the failure. A prognostic biomarker would therefore greatly help future clinical trials in selecting more appropriated participants.

B lymphocyte stimulator (BLyS) and a proliferation-inducing ligand (APRIL) are associated with mechanisms of disease. These proteins are found in plasma and membranes of monocytes, macrophages and dendritic cells. Both can activate B cells and plasma cells to proliferate, differentiate and engage in immunoglobulin G (IgG) class switching [9-11]. Overexpression of BLyS has been shown to cause arthritis and nephritis in mice [12] and by blocking its receptor, one could prevent the development of LN [13]. High serum levels of both proteins are found in SLE patients [14,15]. Furthermore, aberrant expression of both cytokines in memory and plasma cells were tightly correlated with the disease activity score and autoantibody production [16].

Both APRIL and BLyS are found in local tissue like lymph node, bone marrow, synovium and kidney [17-20]. APRIL was also abundant in the cerebrospinal fluid of neuropsychiatric lupus [21]. Since APRIL protein was predominantly found in active lupus nephritis and was locally produced by the glomerular mesangium [22], therefore it may be able to determine the prognosis for lupus nephritis patients. In this study, we assessed the levels of serum and intrarenal APRIL levels in a prospective cohort of active lupus nephritis and its correlation with response to treatment.

## Materials and methods

### Research design

This is a longitudinal study that was conducted from March 2005 through June 2010 at the Lupus Research Unit, Department of Medicine, King Chulalongkorn Memorial Hospital (KCMH). All samples were stored at -80°C until analysis.

The study was approved by the Ethics Committee for Human Research of the Faculty of Medicine, Chulalongkorn University (IRB No. 201/52), and written informed consent was obtained from all patients.

### Study population

All patients who had an SLE classification according to the 1997 criteria established by the American College of Rheumatology (ACR) were recruited from KCMH. Fifty-two patients with active LN were enrolled into the study. The criteria for active LN were urine proteinuria > 0.5 g/day and urine red blood cells (RBC)/white blood cells (WBC) > 5/high power field (HPF) or increased serum creatinine > 0.3 mg/dL [23]. Active LN patients were also required to have a kidney biopsy with an adequate number of glomeruli (more than 10) so the severity of LN could be evaluated.

Twenty-seven patients with biopsy-proven ISN/RPS class III/IV LN were treated with mycophenolate mofetil (MMF) or intravenous cyclophosphamide plus prednisone. Blood samples from these patients were drawn at baseline and six months later. All patients were classified as complete response (CR) or non-CR (partial and non-response) according the ACR criteria [24]. CR means urine protein less than 200 mg per day, urine red blood cells less than 5 per high power field and improved kidney function.

### Data collection

Various data were collected: clinical assessment of SLE Disease Activity Index (SLEDAI), laboratory tests, histological parameters, blood levels of APRIL and BLyS, and measurements of APRIL and BLyS levels from the kidney biopsies. Blood levels of APRIL and BLyS were obtained on the day of the biopsy and on the next six month follow-up visit.

### ELISA measurements for APRIL and BLyS

Enzyme-linked immunosorbent assay (ELISA) was used to quantify levels of APRIL. ELISA plates (R&D Systems, Inc., Minneapolis, MN, USA) were coated with mouse anti-human APRIL polyclonal antibody. Standards and samples were pipetted into the wells. Any APRIL present in the patients' samples were bound by the antibody coated on the plate. After washing the plate for any unbound substances, polyclonal antibodies against APRIL conjugated to horseradish peroxidase were added to the wells. After that, the plate was washed again to remove any unbound antibody-enzyme reagent. Next, a substrate (tetramethylbenzidine) was added to the wells. The plate was incubated at room temperature in the dark for a couple of minutes. When the color changed from blue to yellow, sulfuric acid was added to stop the color development. The amount of APRIL correlated with the intensity of the color. The plate was read at a wavelength of 540 nm or 570 nm.

BlyS was quantitated by the BlyS Platinum ELISA kit (eBioscience, San Diego, CA, USA). The procedure of the ELISA is the same as that used to quantitate the levels of APRIL except for the antibodies used, which would be more specific in detecting BlyS.

For serum APRIL or BLyS levels, measurement was performed in duplication for each samples. The coefficient variation (CV) was calculated and any measurement with CV above 20% was excluded from the analysis. Then, five out of fifty-two samples were excluded due to CV > 20%.

### RNA extraction and cDNA synthesis

RNA was extracted from the renal biopsy specimens of the LN patients. Total RNA was purified by RNeasy purification kit (Qiagen, Valencia, CA, USA). The first strand cDNA was synthesized from 250 ng of total RNA by using MultiScribe™ Reverse Transcriptase enzyme (Applied Biosystems, Carlsbad, CA, USA). The reaction was performed at 45°C for 45 min.

### Quantification of intrarenal mRNA levels of APRIL and BLyS

Intrarenal mRNA levels of APRIL and BLyS were measured by real-time quantitative polymerase chain reaction (RT-PCR). The sequences of the primers are as follows: APRIL sense 5'-AGAGTCTCCCGGAGCAGAGTT-3', APRIL antisense 5'-CTGGTTGCCACATCACCTCTGT-3', BLyS sense 5'-TCACCGCGGGACTGAAAATCTT-3' and BLyS antisense 5'-AGCTGAGAAGCCATGGAACAAATG-3'. Each PCR was carried out in a 20 μL reaction volume composed of 2 μL cDNA template, 10 μL of SYBR Green PCR Master Mix (Applied Biosystems, Carlsbad, CA, USA), 0.5 mM forward primer, and 0.5 mM reverse primer. The amplification reaction was performed by using an Applied Biosystems 7500 Real-Time PCR System (Applied Biosystems, Carlsbad, CA, USA). The comparative Delta Ct method was used to determine the mRNAs levels of APRIL and BLyS. 18s ribosomal RNA was used as the endogenous reference. Dissociation curve analysis confirmed amplification of one specific product per primer pair.

### Renal histology

Paraffin sections were stained with hematoxylin and eosin, periodic acid-Schiff, trichrome, and silver for light microscopy. Activity and severity of the specimens were scored by using the Austin criteria [25]. The maximum scores of the activity and chronicity indices were 24 and 12, respectively. The activity index was calculated by summing the semi-quantitative scores of the following parameters: endocapillary proliferation, fibrinoid necrosis, cellular crescents, leukocyte infiltration, hyaline thrombi and interstitial infiltration. The chronicity index was calculated by adding the semi-quantitative scores of the following parameters: glomerular sclerosis, fibrous crescents, interstitial fibrosis and tubular atrophy. The percentage of each parameter was calculated by the following equation:

$$\text{Percentage of each pathology}=\frac{\text{Number of glomeruli involved }\times 100\%}{\text{Total number of glomeruli obtained}}$$

All biopsies were examined by one pathologist (WK) who was not aware of the molecular results. The histological samples of LN were classified according to the ISN/RPS classification [26].

#### Renal SLEDAI

Renal SLE disease activity index of the SLE patients at the time of visit was measured by using MEX-SLEDAI scoring system [27]. From the MEX-SLEDAI scoring system, the renal disease activity was scored on a 0 to 16 point scale according to the MEX-SLEDAI index. A higher score indicated a greater disease activity. Renal SLEDAI was calculated by summing the scores of the following parameters: urinary casts (heme-granular or red blood cell casts), hematuria (> 5 RBC/HPF by excluding stones, infections, or other causes), proteinuria (> 0.5 gram/24 hours) and pyuria (> 5 WBC/HPF by excluding infections). The maximum score of renal SLEDAI was 16.

#### Data analysis

Statistical analysis was performed by using SPSS software (version 16, SPSS Inc, Chicago, IL, USA). Continuous variables were summarized as mean ± SE or median (interquartile range: IQR) where appropriate and categorical variables were used as frequencies and percentages. Significant differences were calculated by using Mann-Whitney tests for continuous variables. Correlation coefficients were calculated by Spearman's test. Significant factors obtained from univariate analysis were entered into a multiple logistic regression model for control of potential confounding effects. Box plot graphs were done by using GraphPad Prism version 4.03 for Windows (GraphPad Software, San Diego, CA, USA). The receiver operating characteristic curve (ROC) curve of APRIL levels was plotted by using SPSS software. Differences with a *P *value of < 0.05 were considered statistically significant.

For serum APRIL or BLyS levels, measurement was performed in duplication for each sample. The coefficient variation (CV) was calculated and any measurement with CV > 20% was excluded from the analysis.

## Results

### Patient characteristics

All studied patients were biopsy-proven class III or IV by the ISN/RPS classification, which represented common types of renal pathology of the disease. Other histological classes were excluded from this study. The mean age of the patients was 32.9 years. Most of the patients were female (96%). The mean duration of the disease before kidney biopsy was 5.9 years. Median renal SLEDAI score in the active group was 12. Median daily dose of prednisolone was 16 mg. Immunosuppressive drugs mostly used in the study were either oral mycophenolate or pulse intravenous cyclophosphamide. Most of the patients had nephrotic range proteinuria with normal serum creatinine. Levels of serum complements (C3,) were low whereas the antinuclear antibodies (ANA) and anti-dsDNA titers were high. Mean activity and chronicity scores were 7.83 and 3.01, respectively (Table 1). Here, the studied participants clearly represented active lupus nephritis in clinical practice.

**Table 1:** Patient characteristic at baseline.

	Active LN* (*n *= 47)
**Age, years**	
Mean ± SE	32.90 ± 1.29
Range, years	26-39
**Sex, number**	
Female	50 (96)
**Duration of SLE, years**	
Mean ± SE	5.90 ± 0.62
**Renal-SLEDAI score**‡	
Median	12
Range	8-12
**Prednisone use, number **(%)	47 (90)
Median dosage, mg/day	16.00
Dosage range, mg/day	7.5-50
**Immunosuppressive drug use**	
Intravenous methylprednisolone	3
Intravenous cyclophosphamide	6
Mycophenolate mofetil	6
Azathioprine	1
Hydroxychloroquine	2
**Extrarenal manifestation, number (%)**	12 (23)
**BLyS**, ng/ml^a ^	1.32 ± 0.15
**APRIL**, ng/ml^a^	4.10 ± 0.30
Serum creatinine, mg/dl	1.06 ± 0.08
Proteinuria, g/day Urinary erythrocyte count, per high power field	3.59 ± 0.31 50.84 ± 11.96
**Immunological parameters**^a^	
C3 (normal range: 70-140 mg/dL)	60.86 ± 6.05,
ANA, titer	731.20 ± 606.29
Anti-dsDNA, titer	351.74 ± 73.76
**Histological parameters**	
Activity score	7.83 ± 0.70
Chronicity score	3.01 ± 0.37

*Includes only lupus nephritis class III or IV by ISN/RPS 2003 criteria, ‡ renal-SLEDAI (Systemic Lupus Erythematosus Disease Activity Score) uses four endpoints as follows: UPCI or 24-hour urine protein ≥0.5 g/day, urinary RBCs ≥5/HPF, urinary WBCs ≥5/HPF and urinary cellular casts ≥1/HPF; and the maximal point was set at 16. ^a^Data are expressed as mean ± SE. ANA, antinuclear antibody; APRIL, a proliferation-inducing ligand; BlyS, B lymphocyte activation protein; HPF, high power field; LN, lupus nephritis; RBC, red blood cells; SLE, systemic lupus erythematous; WBC, white blood cells.

### The correlation of serum BLyS and APRIL levels with SLE

We studied the stored blood samples drawn at the time of clinically and histologically confirmed active nephritis. The immunosuppressant mostly used were moderate dose of prednisone plus either mycophenolate mofetil or intravenous cyclophosphamide. BLyS levels were significantly correlated with the serum complement level (Rs = 0.46 for C3; *P *value < 0.01), total white blood cell count (Rs = -0.48; *P *value < 0.01), but not anti-dsDNA titer. Moreover, BLyS levels were also associated with dosage of immunosuppressant including steroids and MMF (Rs = 0. 46 and 0.37; respectively; *P *value < 0.01) (Table 2). In a multivariate analysis, MMF was the only factor associated with BLyS levels (*P *< 0.01).

**Table 2:** Correlations of serum levels for BLyS and APRIL to lupus activity parameters, pathological scores, and renal and systemic involvement at baseline.

		BLyS	APRIL
**Renal parameters**			
R_SLEDAI		-0.17	0.22
Serum creatinine	mg/dl	0.06	0.06
Erythrocyturia	cells/high power field	-0.13	0.06
Urine protein	g/day	-0.05	0.44**
Activity score		-0.11	0.34*
Chronicity score		0.25	0.20
			
**Lupus parameters**			
ANA titer		-0.20	0.02
Anti-dsDNA titer		-0.28	0.23
Complement factor 3	mg/dl	0.46**	-0.44**
Total leukocyte count	cells/mm^3^	-0.49***	0.26
Polymorph	cells/mm^3^	-0.40**	0.18
Lymphocyte	cells/mm^3^	-0.42***	-0.14
			
**Treatment**			
Prednisolone	mg/day	0.46***	0.25
Mycophenolate	mg/day	0.37**	-0.09
			

ANA, antinuclear antibody; APRIL, a proliferation-inducing ligand; BlyS, B lymphocyte activation protein; R_SLEDAI, renal Systemic Lupus Erythematous Disease Activity Index. *P < 0.05, **P < 0.01, ***P < 0.001.

**Figure 1:**
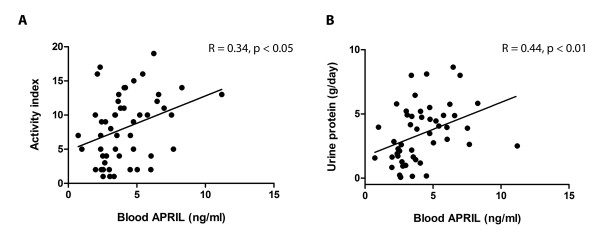
**The correlation between blood levels for APRIL, activity index and 24-hour urine protein**. The relationship between the blood levels for APRIL and renal histology in active LN **(A) **was significantly correlated with a *P *value of < 0.05 (r = 0.34). The blood levels for APRIL and 24-hour urine protein **(B) **were also significantly correlated with *P *value of < 0.01 (r = 0.44).

APRIL levels were significantly correlated with proteinuria (Rs = 0.44; *P *value < 0.01), and renal histology activity index (Rs = 0.34; *P *value < 0.05) (Table 2, Figure 1). These findings emphasize the differential functions of both proteins. While BLyS was influenced by immunosuppressants, APRIL narrowly determined renal-specific disease activity. Since the correlation study was based on a single blood sampling test, the better conclusion would require serial measurement of the cytokines.

### Serum levels of APRIL were positively correlated with the severity of renal histology

Serum levels of APRIL in the active (class III/IV) LN ranged from 0.7 to 11 ng/ml with a mean ± SE of 4.1 ± 0.28 ng/ml. In this active LN study, we found that the serum levels of APRIL were positively correlated to the renal histology results. Serum levels of APRIL were also positively correlated with urine protein (Rs = 0.44; *P *value < 0.01) and renal pathology (Rs = 0.34; *P *value < 0.05) as shown in Figure 1. Cutoff for serum level of APRIL was established by using the mean serum levels of APRIL from the active LN group; patients with high serum levels of APRIL (> 4 ng/ml) had more severe renal histology results as shown in Table 3.

**Table 3:** Serum levels of APRIL and activity score from the renal histological outcomes.

Percentage of glomeruli with the lesions	Blood APRIL≤4 ng/ml	Blood APRIL > 4 ng/ml	*P *value
Endocapillary proliferation	40.99 ± 6.76	64.87 ± 8.6	0.033*
Fibrinoid necrosis	4.69 ± 2.15	12.66 ± 4.1	0.012*
Neutrophil infiltration	16.47 ± 4.85	49.59 ± 8.95	0.004*
Hyaline deposit	22.74 ± 7.37	27.65 ± 7.36	0.609
Cellular crescent	12.38 ± 3.49	15.12 ± 4.27	0.620

*The cutoff level for blood APRIL was 4 ng/ml. This cutoff value was obtained from the average blood levels of APRIL from all patients studied. APRIL, a proliferation-inducing ligand.

### Serum levels of APRIL may predict the patients' responses to immunosuppressive treatment

To determine the prognostic value of serum APRIL and BLyS, a prospective cohort with standard immunosuppressive drugs were studied. Twenty-seven patients with biopsy-proven ISN/RPS class III/IV LN were longitudinally followed and studied after receiving six months of treatment with immunosuppressive drugs. Serum levels of BLyS and APRIL were obtained from baseline and at six months later. In this analysis, we determined the criteria of complete response to treatment by the ACR criteria [24].

**Figure 2:**
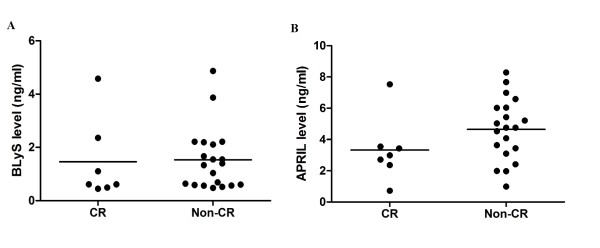
**Comparisons of blood levels for BLyS (A) and APRIL (B) and treatment response**. Each dot represents a baseline serum level of individual patient. CR means complete response to treatment by ACR criteria. Non-CR means partial or non-responses. ACR, American College of Rheumatology; APRIL, a proliferation-inducing ligand; BlyS, B lymphocyte activation protein.

After six months of immunosuppressive therapy, 30% (7 of 27) of the patients achieved complete response (CR). There were no differences in the serum levels of BLyS among the CR and non-CR prior to treatment (Figure 2A). However, the serum levels of APRIL from the CR group were lower when compared to the non-CR group, but had not reached statistical significance (Figure 2B). Six patients from the CR group (86%) had serum levels of APRIL less than 4 ng/ml prior to treatment (Figure 2B). Unlike the CR group, 14/20 (70%) patients from the non-CR group had serum levels of APRIL more than 4 ng/ml prior to treatment. We therefore performed the ROC; we found that the serum levels of APRIL at 4 ng/ml could accurately predict response to treatment with a sensitivity of 65% and a specificity of 87.5%. The calculated area under the ROC curve was 0.713 (95% confidence interval = 0.49 to 0.93). The positive predictive value (PPV) and negative predictive value (NPV) were 93% and 54%, respectively. This cutoff level is the same value as the one obtained earlier from the mean serum levels of APRIL. This indicated that the value selected for the cutoff was appropriate in distinguishing response to treatment.

**Figure 3:**
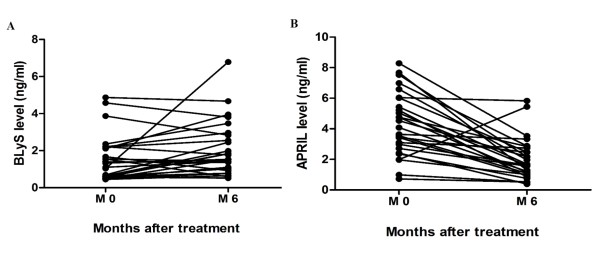
**Changes in blood levels for BLyS and APRIL after six months of treatment in active LN patients**. Dots and bars show blood levels for BLyS **(A) **and APRIL **(B) **in patients with active LN class III or IV at baseline and 180 days posttreatment. APRIL, a proliferation-inducing ligand; BlyS, B lymphocyte activation protein; LN, lupus nephritis.

After six months of immunosuppressive treatment, there was a significant reduction in serum levels of APRIL (4.30 +/- 0.39 ng/mL at baseline, and 2.00 +/- 0.26 ng/mL at sixth month; *P *< 0.001 paired *t *test) while serum BLyS levels slightly increased (1.51 +/- 0.24 ng/mL at baseline, and 2.04 +/- 0.28 ng/mL at sixth month; *P *= 0.04) (Figures 3A and 3B). This finding may represent a balance between the two cytokines.

### Intrarenal levels of APRIL and BLyS could determine the response to treatment

It was interesting to further study intrarenal BLyS and APRIL levels of lupus patients. Renal tissue samples from 36 patients with biopsy-proven class III/IV LN were used to assess the intrarenal levels of APRIL and BLyS. This was done by measuring the levels of mRNA from the renal biopsy samples. The studied samples were divided into two groups according to the patients' response to treatment: responder (*n *= 18) and non-responder (*n *= 18). In this analysis, responder were defined by the following clinical criteria: (1) stable or improved renal function, (2) X50% reduction in hematuria to less than 10 RBC per HPF and (3) significant reduction in proteinuria (X50% reduction to less than 3 g/day if baseline is within the nephrotic range or less than 1 g/day if baseline is not nephrotic) for at least three months.

**Figure 4:**
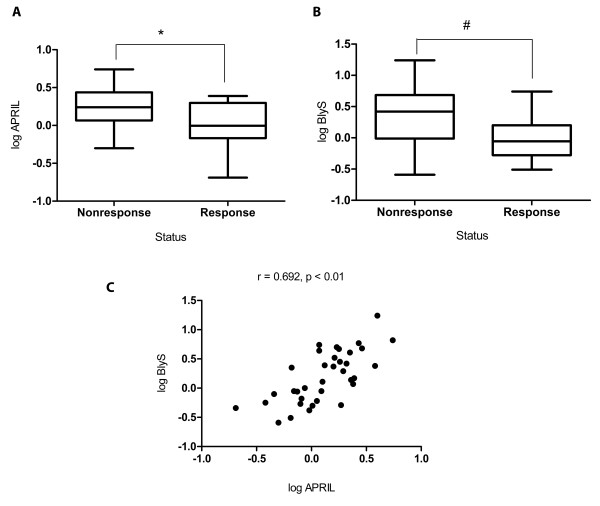
**Correlations of intrarenal mRNA levels for APRIL and BLyS and response to treatment**. Box plots and bars show mRNA levels for APRIL **(A) **and BLyS **(B) **from the renal tissues of the non-responder (*n *= 18) and responder groups (*n *= 18). The mean mRNA levels for APRIL and BLyS from the non-responder group were significantly higher when compared to the patients from the responder group (*P = 0.02, #*P *= 0.007). Dots and bars in **(C) **represent the correlated expression of mRNA levels for BLyS and APRIL in the renal tissue of patients with LN (r = 0.692; *P *< 0.01). APRIL, a proliferation-inducing ligand; BlyS, B lymphocyte activation protein; LN, lupus nephritis.

The non-responder group had significantly higher mRNA levels of APRIL and BLyS compared to the responder group (Figures 4A and 4B). Intrarenal mRNA levels of BLyS were significantly and positively correlated with intrarenal mRNA levels of APRIL as shown in Figure 4C, (r = 0.692; *P *< 0.01). This result suggests that both APRIL and BLyS may play a role in the severity of renal inflammation. Therefore, the intrarenal mRNA levels of APRIL and BLyS may be used to predict the outcome of treatment.

We also studied paired serum and kidney tissues obtained on the same day (*n *= 20). The serum APRIL or BLyS was not associated with its intrarenal levels (data not shown). However, the relationship between circulating and intrarenal APRIL/BLyS warrants further study. Here, we speculate that local factors could also influence intrarenal gene expression.

## Discussion

This study demonstrated that B cell activation markers such as APRIL and BLyS are tightly correlated with SLE and LN disease activity. Serum levels of BLyS were influenced by immunosuppressant whereas serum levels of APRIL could accurately predict the status of the kidney and severity of the disease (Table 2). Patients with high serum levels of APRIL have severe proliferative nephritis such as endocapillary proliferation, fibrinoid necrosis and polymorph infiltration. In addition, serum levels of APRIL more than 4 ng/mL may predict that the patients will experience treatment failure (Table 3 and Figure 2). Moreover, in non-responder patients, intrarenal mRNA levels of APRIL and BLyS were higher compared to the responders (Figure 4). This supported the notion that blocking BLyS and APRIL may be useful in treating lupus nephritis.

BLyS and APRIL have been shown to be involved in B cell-mediated pathology of SLE. In an animal model, it has been shown that BLyS transgenic mice had an elevated number of B cells with prolonged survival times [28]. These transgenic mice exhibited lupus-like phenotypes such as having high titer levels of anti-dsDNA antibody, proteinuria, and glomerulonephritis [28,29]. It has been shown that by blocking the receptors of BLyS and APRIL, TACI-Ig can decrease levels of proteinuria in NZB/W F1 lupus-prone mice [29]. On the other hand, by blocking only BLyS, BAFF-R-Ig has been shown to be able to prolong the lives of NZB/W F1 mice and prevent renal inflammation [30].

These data obtained from the animal studies were consistent with findings in lupus patients. Chu *et al*. [16] found expressions of BLyS and APRIL on CD19+ B cells that were closely correlated with the activities of the disease and autoantibody levels in SLE patients. We also observed an elevated level of circulating BLyS [29], which corresponded to the serologic indices of SLE patients (Table 2). Interestingly, dosages of immunosuppressive drugs affected serum levels of BLyS and APRIL differently (Figure 3A and 3B). Serum levels of both cytokines in this study may release from different B-cell populations [31]. BLyS represents early preimmune B-cell stages and could be easily inhibited by steroids or MMF [32]. In contrast, APRIL may lately respond to the immunosuppressants. We observed high APRIL levels associated with lupus patients without renal involvement and only used low-dose prednisone (data not shown). This was previously demonstrated by Morel *et al*. [33].

Regarding circulating BLyS levels, we observed less serum BlyS levels (1.32 ± 0.15 ng/mL) as compared to other studies (range 1.5 to 19.1 ng/mL) [34]. In contrast to previous published works [34,35], all participants, in this study, were diagnosed lupus glomerulonephritis and had marked proteinuria. As Petri M and Stohl W *et al*. pointed out that lupus nephritis may have a urinary loss of BLyS protein. Therefore, a loss of correlation between circulating BLyS and disease activity may occur in lupus nephritis [34,35]. Unlike other published works, we did not find a correlation between BLyS and anti-dsDNA. This could be explained by the urine loss of BLyS protein. Moreover, potent immunosuppressants (steroids and MMF) may decrease BlyS production while such treatment had less influence on the anti-dsDNA titer [32]. Therefore, different results among published works could be explained by different stages of disease and studied populations.

The utility of biomarkers in clinical nephrology has been successfully demonstrated by leaders in the field [36,37]. Since serum APRIL levels were associated with the histology of nephritis, and high serum levels (> 4 ng/mL) of APRIL may predict treatment failure in patients with class III/IV LN on standard course of therapy. Thus, levels of APRIL may be used as a biomarker to prognose or guide the treatment for LN. This would indicate that patients with class III/IV LN and high serum levels of APRIL will need additional therapy in conjunction with the standard immunosuppressive treatment in order to successfully treat the disease.

The use of monoclonal antibodies such as belimumab together with standard therapy has been shown to work in treating patients with mild forms of SLE without renal or neurological organ involvement [38-40]. Although belimumab is the only monoclonal antibody that has already been approved to treat SLE, monoclonal antibodies to APRIL/BLyS are ongoing in clinical trials. Current treatment for severely active LN are only limited to immunosuppressants such as steroids, cyclophosphamide or mycophenolate. For non-responsive LN patients, they will inevitably progress to end-stage kidney disease.

Currently, there are clinical trials assessing the safety and efficacy of monoclonal antibodies to BLyS/APRIL such as atacicept. The recombinant protein, atacicept, was studied in phase II/III trial together with MMF and steroid in newly active LN. Unfortunately, patients who received atacicept had profound hypogammaglobulinemia and infection [41]. Combination use of atacicept with MMF is therefore prohibited. In order to sufficiently evaluate the safety and efficacy of these antibodies, selection of participants with active LN is extremely crucial. Based on the results obtained from our study, we postulate that LN patients with high serum levels of APRIL may benefit from using atacicept or other targeted APRIL therapy.

Equally important, our study has shed some light on the pathogenesis of LN. We postulate that local factors may control these gene expressions. BLyS/APRIL mRNAs and proteins were previously observed in the glomerulus and inflammatory infiltrating cells [22]. This would indeed explain why our patients with high intrarenal mRNAs were resistant to treatment. Excess levels of APRIL and BLyS may promote survival of autoreactive B cells in the kidney, causing an increased production of pathogenic autoantibodies, and finally resulting in immune-complex-induced glomerulonephritis.

There were some limitations encountered in this study. Aside from the rather small sample size in this study, the studied samples were collected from patients who received different immunosuppressive therapies. Therefore, a further validation study on samples collected before initiation of immunosuppressant should be done in a prospective clinical trial. Nonetheless, this study clearly showed that the B cell activation biomarkers were present in the circulation and kidney tissues of active lupus nephritis patients.

## Conclusions

This study supported the pivotal role for both soluble and tissue levels of APRIL and BLyS in lupus nephritis patients. Since serum levels of APRIL were specific to renal involvement, it may be used as a non-invasive prognostic biomarker for severe LN. Blocking APRIL may be a new way to treat patients with difficult-to-treat LN.

## Abbreviations

ACR: American College of Rheumatology; ANA: antinuclear antibody; APRIL: a proliferation-inducing ligand; BlyS/BAFF: B lymphocyte activation protein; CR: complete response; ELISA: enzyme-linked immunosorbent assay; HPF: high power field; LN: lupus nephritis; MMF: mycophenolate mofetil; NPV: negative predictive value; PPV: positive predictive value; RBC: red blood cells; ROC: receiver operating characteristic curve; RT-PCR: reverse transcription polymerase chain reaction; SLE: systemic lupus erythematous; SLEDAI: SLE Disease Activity Index; WBC: white blood cells.

## Competing interests

The authors declare that they have no competing interests.

## Authors' contributions

WT and PT performed experiments, analyzed data, and wrote the manuscript. TB and PS collected the samples and performed the ELISA. WK reviewed and interpreted the kidney histological results. SE and AL reviewed the manuscript. NH and YA led the project, reviewed the clinical data, provided patient care, and finalized the manuscript. All authors have read and approved the manuscript for publication.
